# The genes and enzymes of the carotenoid metabolic pathway in *Vitis vinifera* L.

**DOI:** 10.1186/1471-2164-13-243

**Published:** 2012-06-15

**Authors:** Philip R Young, Justin G Lashbrooke, Erik Alexandersson, Dan Jacobson, Claudio Moser, Riccardo Velasco, Melané A Vivier

**Affiliations:** 1 Institute for Wine Biotechnology, Department of Viticulture and Oenology, Stellenbosch University, Matieland, 7602, South Africa; 2 Department of Plant Protection Biology, Swedish University of Agricultural Sciences, SE-230 53, Alnarp, Sweden; 3 Genomics and Biology of Fruit Crops Department, IASMA Research and Innovation Centre, Fondazione Edmund Mach Via E. Mach 1, San Michele all'Adige, 38010, , TN, Italy

## Abstract

****Background**:**

Carotenoids are a heterogeneous group of plant isoprenoids primarily involved in photosynthesis. In plants the cleavage of carotenoids leads to the formation of the phytohormones abscisic acid and strigolactone, and C_13_-norisoprenoids involved in the characteristic flavour and aroma compounds in flowers and fruits and are of specific importance in the varietal character of grapes and wine. This work extends the previous reports of carotenoid gene expression and photosynthetic pigment analysis by providing an up-to-date pathway analysis and an important framework for the analysis of carotenoid metabolic pathways in grapevine.

****Results**:**

Comparative genomics was used to identify 42 genes putatively involved in carotenoid biosynthesis/catabolism in grapevine. The genes are distributed on 16 of the 19 chromosomes and have been localised to the physical map of the heterozygous ENTAV115 grapevine sequence. Nine of the genes occur as single copies whereas the rest of the carotenoid metabolic genes have more than one paralogue. The cDNA copies of eleven corresponding genes from *Vitis vinifera* L. cv. Pinotage were characterised, and four where shown to be functional. Microarrays provided expression profiles of 39 accessions in the metabolic pathway during three berry developmental stages in Sauvignon blanc, whereas an optimised HPLC analysis provided the concentrations of individual carotenoids. This provides evidence of the functioning of the lutein epoxide cycle and the respective genes in grapevine. Similarly, orthologues of genes leading to the formation of strigolactone involved in shoot branching inhibition were identified: *CCD7*, *CCD8* and *MAX1*. Moreover, the isoforms typically have different expression patterns, confirming the complex regulation of the pathway. Of particular interest is the expression pattern of the three *VvNCEDs*: Our results support previous findings that *VvNCED3* is likely the isoform linked to ABA content in berries.

****Conclusions**:**

The carotenoid metabolic pathway is well characterised, and the genes and enzymes have been studied in a number of plants. The study of the 42 carotenoid pathway genes of grapevine showed that they share a high degree of similarity with other eudicots. Expression and pigment profiling of developing berries provided insights into the most complete grapevine carotenoid pathway representation. This study represents an important reference study for further characterisation of carotenoid biosynthesis and catabolism in grapevine.

## **Background**

In the last decade genomic research has been characterised by the initiation and completion of a number of sequencing projects from a diverse array of genus/species, including grapevine. With two grapevine genome sequences completed [1,2] the non-trivial task of identifying and assigning biological functions to the putative gene assignments begins [3]. The availability of sequenced genomes and comparative genomics currently makes it possible to identify putative orthologues quite rapidly [4,5]. The annotation of unknown proteins with unclear functions presents a significant challenge and potentially holds unique/novel information specifically for grapevine research. This study targeted the carotenoid biosynthetic/catabolic pathways of grapevine to provide a baseline understanding of the genes, their genomic organisation and their expression patterns in developing berries. Previous studies reporting on genome organization and/or expression patterns in grapevine relied on ESTs, partial genome sequences or candidate genes. The carotenoid metabolic pathway is highly conserved and ubiquitous in photosynthetic organisms. (reviewed in [6]). Carotenoids are essential pigments in photosynthetic organisms (plants and some micro-organisms) and are involved in a number of physiological and developmental processes. In plants, carotenoids accumulate in leaves, flowers and fruits; and their major function is the protection of the photosynthetic membranes. The enzymatic or oxidative cleavage of carotenoids leads to the formation of apocarotenoids with functions ranging from phytohormones (i.e. abscisic acid and strigolactone) to volatile flavour/aroma compounds (e.g. β-ionone, β-damascenone and trimethyl- dihydro-naphtalene (TDN) (reviewed in [7-9]. The carotenoid content of plants has also received attention due to their antioxidant- and provitamin A potential and thus their importance to both animal and human nutrition [10,11]. The genes involved in carotenoid biosynthesis and catabolism are therefore attractive targets for genetic manipulation to increase the carotenoid content of fruits and seeds. A number of papers have been published reporting the successful genetic modification of crop plants and includes rice, tomato, potato and canola [12-15].

Comparative genomics was used to identify 42 putative orthologues in the grapevine genomic sequence for all the known enzymatic reactions in the carotenoid biosynthetic and catabolic pathways of plants. Grape-specific arrays were used to profile the expression of the annotated genes in the carotenoid biosynthetic/catabolic pathways during three distinct stages of berry development: green, véraison and ripe/harvest stages and the expression correlated to experimentally obtained berry carotenoid concentrations. Pathway analysis (both biosynthetic and catabolic) of the respective genes and metabolites was used to follow carotenoid evolution during berry development in *Vitis vinifera* L. cv Sauvignon blanc. The corresponding cDNA copies of 11 of the 42 orthologues identified have been isolated, sequenced and additionally four have been shown to be functional and are discussed in the broader context of carotenoid biosynthesis.

## **Results**

### ***In silico*characterisation of the carotenoid metabolic pathway genes from*Vitis vinifera***

**Figure 1:**
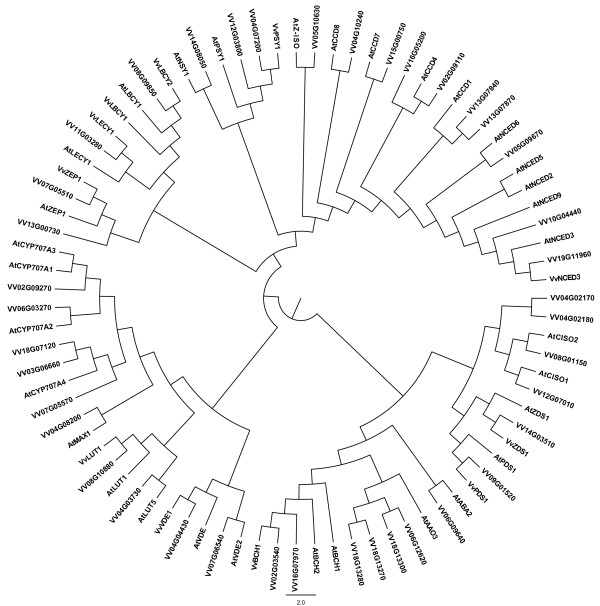
**Molecular phylogenetic tree of the carotenoid biosynthetic/catabolic genes from *Arabidopsis thaliana *(At-) and the identified *Vitis vinifera *(Vv-) orthologues listed in Additional file ****2****: Table SM1.** The tree was generated using PhyML with default parameters.

Forty two *V. vinifera* putative carotenoid metabolic gene orthologues were identified by *in silico* screening of the PLAZA [4,5] grapevine database using the *Arabidopsis* sequences obtained from AtIPD [16]. A multiple alignment was created using the 42 *Vitis* sequences and the corresponding *Arabidopsis* orthologues and a maximum likelihood method [17] used to generate a bootstrapped molecular phylogenetic tree (Figure 1). Sequence similarity was used to assign putative function(s) to the identified grapevine orthologues. Additional file 1 lists the grapevine gene name, the corresponding PLAZA accession(s), the associated Roche Nimblegen probe accession, the *Arabidopsis* orthologue accession and the putative gene assignment.

Additional file 2 shows the distribution of the 37 (of the 42) genes identified and listed in Additional file 1 in the grapevine genome. Where possible the genes were positioned on the genome sequence assembly of the heterozygous ENTAV115 described in Velasco et al. [1]. Five of the genes could not be localised on the ENTAV115 genome sequence, but were localised in the PN40024 genome sequence. The putative genes are shown on the corresponding linkage groups together with reference markers of the genetic map described in Troggio et al. [18]. The carotenoid metabolic genes are distributed throughout the grapevine genome, and are positioned on 16 of the 19 chromosomes (all except chromosomes 1, 9 and 17 contain pathway members). Two tandem duplications are evident amongst the genes analysed: *VvPDH1* and *VvPDH2* on chromosome 4, and *VvCCD1.1* and *VvCCD1.2* on chromosome 13. Two segmental duplication events are also present: *VvCYP707A2.2* on chromosome 7 and *VvCYP707A2.3* on chromosome 18, as well as *VvBCH1* on chromosome 2 and *VvBCH2* on chromosome 16.

### **Isolation and characterisation of 11 of the carotenoid metabolic pathway genes from*Vitis vinifera***

The full-length cDNA copies of 11 of the 42 genes listed in Additional file 1 were PCR-amplified from V. vinifera L. cv. Pinotage cDNA, namely: *VvPSY1*, -*PDS1*, -*ZDS1*, -*LECY1*, -*LUT1*, -*LBCY2*, *LBCY1*, -*BCH1*, -*ZEP1*, -*VDE1*, and –*NCED3*. The cDNA fragments were isolated, cloned and verified by sequencing and submitted to Genbank: *VvPSY1* (JQ319634), -*PDS1* (JQ319635), -*ZDS1* (JQ319636), -*LECY1* (JQ319637), -*LUT1* (JQ319638), -*LBCY2* (JQ319639), *LBCY1* (JQ319643), -*BCH1* (JQ319640), -*ZEP1* (JQ319641), -*VDE1* (JQ319642), and –*NCED3* (JQ319644). Additional file 1 lists the genes, sequence information and the closest *Arabidopsis* match in the publicly available grapevine sequence repositories.

The ProtComp server [19] was used to predict the sub-cellular localisation of the respective predicted proteins (of the isolated genes) (Table 1). These results indicated that the predicted proteins of the *V. vinifera* genes are likely localised in the chloroplast, as found in other species [6]. The respective genomic sequences of the isolated carotenoid metabolic genes were identified based on homology to the cDNA sequences. Table 1 lists the sizes of the full-length cDNA- and genomic copies of the isolated grapevine genes as well as the associated linkage groups. Analysis of the genomic structure of a subset of the carotenoid metabolic genes (listed in Additional file 1) showed that the number of exons of the isolated carotenoid metabolic genes in the available plant genomes is remarkably well conserved (Table 2). The degree of identity between the predicted amino acid sequence for each isolated grapevine carotenoid biosynthetic/catabolic genes and the respective orthologues in *A. thaliana, P. trichocarpa* and *O. sativa* was calculated. The amino acid sequences of the grapevine genes are highly conserved and phylogenetically more similar to members of the eudicots (e.g. *Arabidopsis* and *Populus*) than the monocots (e.g. *Oryza* and *Sorghum*) (Table 3).

**Table 1:** Sequence information and related accessions

**GENE**	**cTP**^**1**^	**cDNA (bp)**^**2**^	**gDNA (bp)**^**3**^	**DFCI TIGR** **Accession**^**4**^	**Unigene Accession**^**5**^	**Associated Linkage Group (LG)**^**6**^	
						**IASMA**^**7**^	**Genoscope**^**8**^
*VvPSY1*	YES	1317	3837	TC43355	Vvi.4169	LG4	LG4
*VvPDS1*	YES	1749	26291	CN007512	Vvi.17887	ND^9^	LG9
*VvZDS1*	YES	1752	12807	TC4818	Vvi.6755	LG14	LG14
*VvLECY1*	YES	1593	6670	TC49056	Vvi.11880	LG11	LG11
*VvLUT1*	YES	1692	5244	EC943050	None^10^	LG8	LG8
*VvLBCY2*	YES	1515	1515	TC50860	Vvi.2624	LG8	LG8
*VvLBCY1*	YES	1494	1494	EC925449	Vvi.18979	LG6	LG6
*VvBCH1*	YES	900	1660	TC42069	Vvi.2348	LG2	LG2
*VvZEP1*	YES	1977	7524	TC46508	Vvi.1307	LG7	LG7
*VvVDE1*	YES	1440	4971	TC47195	Vvi.1247	LG4	LG4
*VvNCED3*	YES	1833	1833	TC48377	Vvi.509	LG19	LG19

^1^ cTP: Softberry ProtComp (http://www.softberry.com/berry.phtml) prediction for a chloroplast transit peptide.

^2^ The size in base pairs of the cDNA copy of the gene from the predicted ATG to the predicted STOP codon;

^3^ The size in base pairs of the genomic copy of the gene from the predicted ATG to the predicted STOP codon;

^4^ The DFCI TIGR (http://www.tigr.org) accession number(s) of tentative consensus sequence corresponding to the isolated gene;

^5^ The Unigene (http://www.ncbi.nlm.nih.gov/unigene) accession numbers(s) corresponding to the isolated gene;

^6^ The grapevine linkage group (LG) associated with the isolated gene;

^7^ The heterozygous ENTAV115 Pinot noir genome sequence from IASMA (http://genomics.research.iasma.it/iasma/);

^8^ The homozygous PN40024 Pinot noir genome sequence from Genoscope (http://www.cns.fr/spip/Vitis-vinifera-whole-genome.html);

^9^ ND: Not determined; could not be mapped to the ENTAV115 genomic sequence.

^10^ None: No associated *Vitis* Unigene(s) (http://www.ncbi.nlm.nih.gov/UniGene/UGOrg.cgi?TAXID=29760).

**Table 2:** **Percentage identity of the predicted protein sequences of the carotenoid metabolic genes (expressed relative to the *Vitis vinifera *orthologue) **

	**Accession number**^**1 **^ **Percentage identity**^2^** relative to the *Vitis vinifera *orthologue**			
**GENE**	***Vitis vinifera***	***Arabidopsis thaliana***	***Populus trichocarpa***	***Oryza sativa***
**VvBCH1**	VV02G00220 100%	AT4G25700 66.24%	PT00G07815 66.67%	OS03G03370 68.04%
				
**VvECH1**	VV08G13950 100%	AT3G53130 76.68%	PT00G01240 80.33%	OS10G39930 69.91%
				
**VvLBCY2**	VV08G15130 100%	AT3G10230 79.49%	PT16G03200 89.09%	OS02G09750 69.67%
				
**VvLBCY1**	VV00G44920 100%	No orthologue	PT09G01040 76.91%	No orthologue
				
**VvLECY1**	VV11G01840 100%	AT5G57030 76.13%	PT06G09650 84.16%	OS01G39960 68.68%
				
**VvNCED3**	VV19G09570 100%	AT3G14440 71.64%	PT11G07980 79.39%	OS03G44380 64.43%
				
**VvPDS1**	VV09G00040 100%	AT4G14210 81.66%	PT14G09510 84.74%	OS03G08570 77.15%
				
**VvPSY1**	VV00G37410 100%	AT5G17230 71.14%	PT00G07390 87.32%	OS06G51290 68.78%
				
**VvVDE1**	VV00G14320 100%	AT1G08550 66.02%	PT13G04830 86.75%	OS04G31040
				
**VvZDS1**	VV14G05860 100%	AT3G04870 82.72%	PT00G12105 89.62%	OS07G10490 80.10%
				
**VvZEP1**	VV07G11310 100%	AT5G67030 71.41%	PT05G05140 72.73%	OS04G37619 69.30%
				

^1^ Accession from Plaza 1.0 (April 2009): http://bioinformatics.psb.ugent.be/plaza_v1/.

^2^ Percentage identity calculated by pairwise alignments of protein sequences using Jalview.

**Table 3:** The carotenoid metabolic genes: Gene families and the conservation of exon number

			***Vitis***	***Arabidopsis***	***Populus***	***Oryza***	
			***vinifera***^**1**^	***thaliana***^**2**^	***trichocarpa***^**3**^	***sativa***^**4**^	
			**Accession**	**Accession**	**Accession**	**Accession**	**Average**
			**Exon (intron)**^**5**^	**Exon (intron)**	**Exon (intron)**	**Exon (intron)**	**Exon number**
**Gene**	**Gene family**^**6**^	**Sub family**^**7**^					
PSY	HOMO01375	ORTHO001026	VV00G37410	AT5G17230	PT00G07390	OS06G51290	6
			6 (5)	6 (5)	6 (5)	6 (5)	
PDS	HOM001528	ORTHO003376	VV09G00040	AT4G14210	PT14G09510	OS03G08570	14
			14 (13)	14 (13)	14 (13)	14 (13)	
ZDS	HOM001528	ORTHO003216	VV14G05860	AT3G04870	PT00G12105	OS07G10490	14 ± 0.8
			14 (13)	15 (14)	14 (13)	14 (13)	
CISO	HOM003569	-	VV08G02490	AT1G06820	PT16G05590	OS11G36440	13
			13 (12)	13 (12)	13 (12)	13 (12)	
LECY	HOM001393	ORTHO004775	VV11G01840	AT5G57030	PT06G09650	OS01G39960	9.5 ± 2.4
			11 (10)	11 (10)	6 (5)	10 (9)	
ECH	HOM000057	-	VV08G13950	AT3G53130	PT00G01240	OS10G39930	9.3 ± 0.5
			9 (8)	9 (8)	9 (8)	10 (9)	
LBCY	HOM001393	ORTHO005684	VV08G15130	AT3G10230	PT16G03200	OS02G09750	1
			1 (0)	1 (0)	1 (0)	1 (0)	
BCH	HOM002126	-	VV02G00220	AT4G25700	PT00G07815	OS03G03370	6.8 ± 0.5
			7 (6)	7 (6)	7 (6)	6 (5)	
ZEP	HOM004000	-	VV07G11310	AT5G67030	PT05G05140	OS04G37619	16
			16 (15)	16 (15)	16 (15)	16 (15)	
VDE	HOM005780	-	VV00G14320	AT1G08550	PT13G04830	OS04G31040	4.3 ± 1.5
			5 (4)	5 (4)	2 (1)	5 (4)	
NSY	HOM001393	ORTHO015350	VV00G44920	NA	PT09G01040	NA	1
			1 (0)		1 (0)		
NCED	HOM000257	ORTHO000562	VV19G09570	AT3G14440	PT11G07980	OS03G44380	1
			1 (0)	1 (0)	1 (0)	1 (0)	
CCD	HOM000257	ORTHO003354	VV13G12530	AT3G63520	PT01G17700	OS12G44310	14
			14 (13)	14 (13)	14 (13)	14 (13)	

^1^*Vitis vinifera* sequence data from Genoscope v1: http://grup.bio.unipd.it/index.php/Grape_Genome.

^2^*Arabidopsis thaliana* sequence data from TAIR8: http://www.arabidopsis.org/.

^3^*Populus trichocarpa* sequence data from JGI 1.1: http://genome.jgi-psf.org/Poptr1_1/Poptr1_1.home.html.

^4^*Oryza sativa* sequence information from TIGR5: http://rice.plantbiology.msu.edu/.

^5^ Accession number from Plaza 1.0 (April 2009): (PLAZA v1: http://bioinformatics.psb.ugent.be/plaza_v1/).

^6^ The gene family that the homologues are group into (PLAZA v1: http://bioinformatics.psb.ugent.be/plaza_v1/).

^7^ The sub-family that the orthologues group into (PLAZA v1: http://bioinformatics.psb.ugent.be/plaza_v1/).

### **Functional analysis of VvPSY1, VvLECY1, VvLBCY2 and VvBCH1**

The functionality of the isolated VvPSY1, VvLECY1, VvLBCY2 and VvBCH1 was assayed using a bacterial complementation system (described in [20,21]. The pigments accumulated in the transformed *E. coli* cells were extracted and analysed by HPLC and the characteristic formation/degradation of pigments used to determine the functionality of the expressed plant genes. The grapevine VvPSY1, a putative phytoene synthase, could functionally complement a bacterial phytoene synthase in the heterologous bacterial assay (Table 4). VvLECY1, a putative lycopene ε-cyclase converted 42.1% of the available lycopene to ζ-carotene; VvLBCY2, a putative lycopene β-cyclase, converted 72.1% of the available lycopene to β-carotene; whereas VvBCH1, a putative β-carotene hydroxylase, converted 94.5% of the available β-carotene to β-cryptoxanthin (26.9%) and zeaxanthin (67.6%) (Table 4).

**Table 4:** **Functional complementation of grapevine carotenoid biosynthetic genes in *Escherichia coli ***

**Strain**	**Lycopene**	**δ-carotene**	**β-carotene**	**β-cryptoxanthin**	**zeaxanthin**	**Unknown**	**Total pigments**
pAC-LYC + pGEM (control)	100.00	ND^1^	ND	ND	ND	ND	100.00
pAC-LYC + VvLBCY2	22.4	ND	72.1	ND	ND	5.5	100.00
pAC-LYC + VvLECY1	57.90	42.10	ND	ND	ND	ND	100.00
pAC-BETA + pGEM (control)	ND	ND	100.00	ND	ND	ND	100.00
pAC-BETA + VvBCH1	ND	ND	5.5	26.9	67.6	ND	100.00
pAC-85b + pGEM (control)	ND	ND	ND	ND	ND	ND	ND
pAC-85b + VvPSY1	ND	ND	100.00	ND	ND	ND	100.00

^1^ “ND”, not detected.

Carotenoid accumulating *E. coli* cells were transformed with the respective cloned cDNA copies of the grapevine genes. The individual carotenoids analysed are expressed as percentages of the total pigments formed (standard error ≤ 5% (n ≥ 3).

### **Expression analysis of the 42 carotenoid metabolic genes in developing*Vitis vinifera*L. Cv Sauvignon blanc berries**

**Figure 2:**
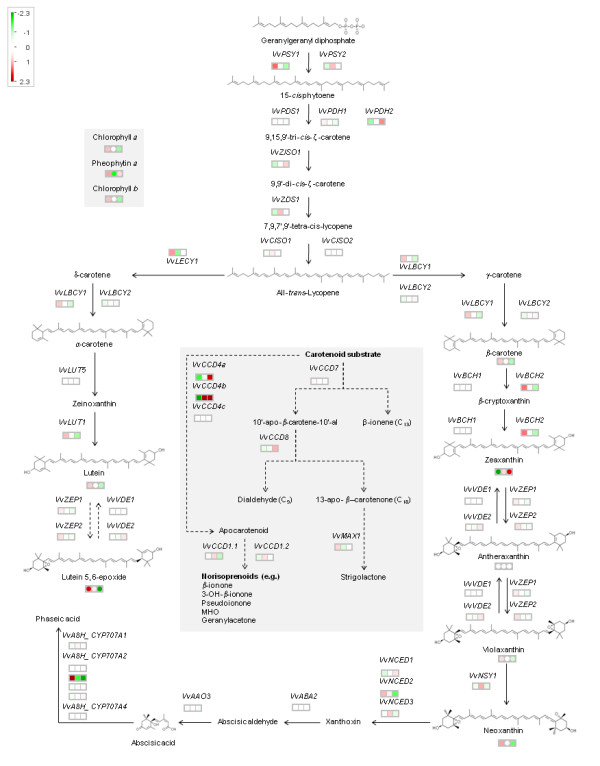
**Pathway analysis of the carotenoid biosynthetic and catabolic pathways.** A Mapman heat map representation of the relative changes in gene and metabolites levels at the three stages of berry development (E-L stage 31, -34 and −38). The values for the transcripts (squares) and carotenoids (circles) have been log2-scaled and mean centred. The amplitude of the carotenoid values are scaled up 100x for visualisation. Refer to Additional file 3: Table SM2 (expression values) and Additional file 5: Table SM3 (carotenoid concentrations) for the absolute values.

**Figure 3:**
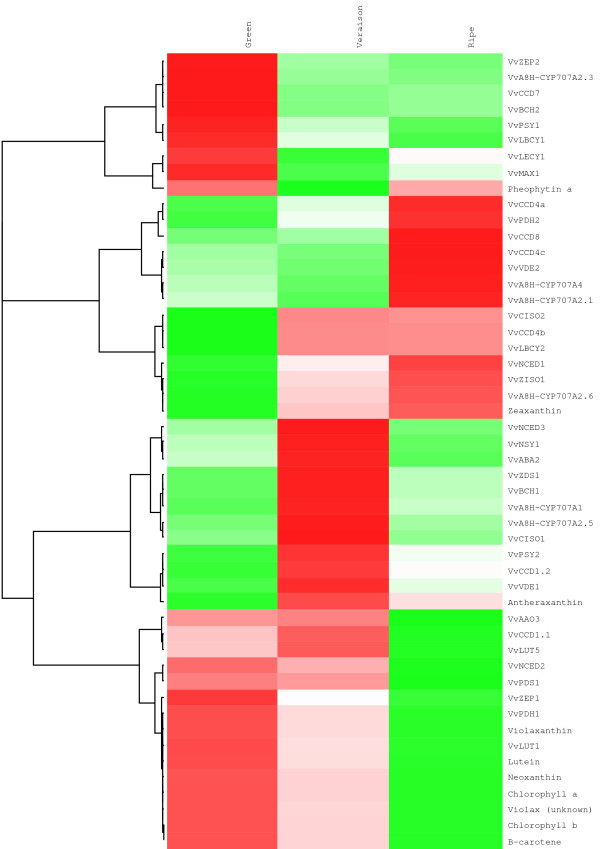
Hierarchical cluster analysis of the expression and metabolites of the carotenoid biosynthetic and catabolic pathway.

The expression of the 42 carotenoid biosynthetic/catabolic genes listed in Additional file 1 was analysed by Nimblegen microarrays (12 × 135 K). The expression data (Additional file 3 is represented in Figure 2 as a heat map in the context of the individual gene’s position within the carotenoid metabolic pathway). Cluster analysis was used to identify and group the carotenoid metabolic genes having similar developmental expression patterns (Figure 3).

From Figures 2 and 3 it can be seen that the expression of six of the carotenoid metabolic genes increased throughout the berry developmental stages: *VvPDH1, VvZISO1, VvLBCY2, VvVDE2, VvCCD4a* and *VvCCD4b.* The expression of *VvCCD4b* increased dramatically from relatively low levels in the green stage reaching ~256-fold upregulation by vérasion and ~315-fold by ripe/harvest stage (relative to the green stage). Similarly, but to a lesser extent, *VvCCD4a* shows a 13-fold upregulation at the ripe/harvest stage (relative to the initial transcript levels at the green stage). Conversely the expression of ten genes decreases as the season progresses: *VvA8H-CYP707A2.1, VvBCH2, VvLBCY1, VvLUT1, VvNCED2, VvPDH1, VvPDS1, VvPSY1, VvZEP1* and *VvZEP2. VvA8H-CYP707A2.1* displays the most downregulation, reaching a 70-fold decrease from the green to the ripe/harvest stage. Similarly, the expression of *VvBCH2, VvLUT1, VvPSY1* and *VvNCED2* is downregulated 4-6-fold by the ripe/harvest stage (relative to the green stage). The expression profiles of a number of genes change at or around the véraison stage. For *VvA8H-CYP707A1, VvCCD1.1, VvCCD1.2, VvCISO1, VvNCED3, VvNSY1, VvPSY2, VvVDE1* and *VvZDS1* the expression peaks at véraison; and for *VvLECY1*, *VvMAX1* and *VvVDE2* the expression is lowest at véraison and increases up until the ripe/harvest stage. Absolute expression values were constitutively low for *VvA8H-CYP707A2_3, VvA8H-CYP707A4, VvABA2, VvBCH1, VvCCD4c VvCCD7* and *VvCCD8* in the berry stages investigated, and transcriptional regulation of these genes may be tissue-specific and/or developmental regulated. VvBCH1 was, however, shown to be functional in a heterologous complementation system (Table 4).

Only *VvZDS1, VvZISO1, VvABA2, VvLECY1, VvLUT1, VvLUT5, VvNSY1, VvCCD7* and *VvCCD8* occur as single copy genes in the grapevine genome; the rest of the carotenoid metabolic genes have more than one paralogue (isoforms). The different isoforms of the carotenoid metabolic genes typically have distinctly different expression patterns (Figure 2 and Figure 3). For example, the expression profiles of the three putative *VvPDS/PDH*-encoding genes have three distinct expression patterns: *VvPDS1* and *VvPDH1* have similar profiles with expression decreasing as the growth season progresses. *VvPDH1*, however, has an inverse expression pattern and increased, peaking at harvest. *VvZEP1* and *VvZEP2* are an exception and have a similar expression pattern (both profiles decreased from the green to the ripe/harvest stage) (Figure 2).

Four groupings of genes are of particular interest in the pathway representation in Figure 2: (1) the genes encoding for enzymes involved in the violaxanthin- and lutein epoxide (xanthophyll) cycles involved in photoprotection (*VvVDE1, VvVDE2, VvZEP1* and *VvZEP2*); (2) genes encoding for enzymes that cleave carotenoids to form the phytohormone ABA (*VvNCED1, VvNCED2* and *VvNCED3*); (3) genes encoding for enzymes that cleave carotenoids to form the phytohormone strigolactone (*VvCCD7, VvCCD8* and *MAX1*); and (4) genes encoding for enzymes that cleave carotenoids to form volatile flavour- and aroma-related compounds e.g. β-ionone *(VvCCD1, VvCCD4a* and *VvCCD4b)*[22-24].

### **Carotenoids and chlorophylls in developing Sauvignon blanc berries**

The photosynthetic pigments (chlorophylls and carotenoids) were monitored at the three sampling time-points. Additional file 4 and Figures 2 and 3 show the changes in carotenoid and chlorophyll concentrations during ripening in the grape berries. The majority of the photosynthetic pigments (chlorophyll a and b, β-carotene, lutein, violaxanthin and neoxanthin) decreased throughout ripening (i.e. from the early/green stage till the ripe/harvest date). The total carotenoid content decreases approximately 3-fold during ripening/maturation (Additional file 4). This trend can be mainly attributed to decreases in lutein and β-carotene that represent the most abundant carotenoids in berries (representing 55-60% of the total carotenoid pool size). Interestingly, the xanthophylls zeaxanthin and antheraxanthin showed an inverse trend by increasing, with zeaxanthin peaking at harvest (E-L stage 38); and antheraxanthin peaking at véraison (E-L stage 34). The ratio of carotenoids/chlorophylls (~0.2) as well as the ratio of chlorophyll a/chlorophyll b (~1.7) remains relatively constant throughout the sampling stages.

### **Abscisic acid levels in developing Sauvignon blanc berries**

ABA was measured at the three sampling time-points. Additional file 5 shows the changes in ABA levels during ripening in the grape berries. The skin and pulp (without seeds) were analysed collectively. ABA concentration increased from the initial green stage, peaking at véraison. ABA levels increase ~5-fold from the green to véraison stage, and decreases ~4-fold from the véraison stage to the ripe/harvest stage.

## **Discussion**

### **Distribution of the carotenoid metabolic genes in the grape genome**

The number of grapevine cultivars is estimated at between 5,000 and 8,000. The availability of this relatively large germplasm collection, and the fact that crosses between wild and domesticated grape species result in fertile hybrids; makes the use of marker assisted selection (MAS) particularly useful for breeders. MAS allows the monitoring of segregation patterns of specific markers in the progeny, as well as the potential to identify genotypes with multiple loci at a relatively early stage.

Gene targeted markers (or functional markers) are especially useful for the MAS approach because in this case the marker coincides with the gene sequence and it is strictly associated with the trait of interest, if known. An example of the potential of this approach in grapevine has been reported by Battilana and co-workers [25]. They demonstrated that the *DXS* gene (encoding a 1-deoxy-d-xylulose 5-phosphate synthase) is the major determinant of monoterpene content in the grapes by QTL analysis and candidate gene approach. Similarly, linking the genes of the carotenoid metabolic pathway to a viticultural/oenological quality parameter can facilitate targeted marker-assisted (molecular) breeding.

Here we show that the carotenoid metabolic genes are distributed on 17 of the 19 grapevine chromosomes (Additional file 2) and we identified the flanking molecular markers. Moreover, through functional complementation assays in bacteria, we confirm the functions of VvPSY1, VvLECY1, VvLBCY2 and VvBCH1, thus providing functional markers for carotenoid metabolism (Additional file 2). The system showed conversion of the substrates to form the products shown in Table 4, confirming the usefulness of this bacterial system for heterologous expression of plant carotenoid metabolic genes. The reasons for the partial or incomplete conversion observed in the functional complementation assay were not investigated, but is possibly due to the difficulties experienced when expressing plant (eukaryotic) proteins in bacterial (prokaryotic) systems.

The grapevine orthologues involved in the formation of the phytohormone strigolactone: *VvCCD7, VvCCD8* and *VvMAX1* have been identified. The absolute expression values of all three genes remained relatively low in the berry stages analysed (Figure 2). Whether or not these genes are transcriptionally active in other plant organs (i.e. the roots) is currently not known and requires further study.

The *in silico* analysis showed conservation of the number of exons of the isolated carotenoid biosynthetic/catabolic genes across plant species. This phenomenon has previously been observed in carotenoid metabolic genes in both the maize and rice phytoene desaturase encoding (*PDS*) genes [26] and to a lesser extent in the CCD gene family (*CCD1, CCD4a, CCD4b, CCD7 and CCD8*) [27]. The reason for the observed conservation of intron number/density/abundance is unclear but the phenomenon seems quite common. For example, a study comparing 117 human and mouse orthologous genes found that 95% of these genes had the same number of exons [28].

### **Carotenoid biosynthesis and catabolism in developing Sauvignon blanc berries**

Grapevine berry development and ripening is a relatively well-studied process. The carotenoid content of grapevine berries has similarly received much attention and typically a steady decline in the abundance of carotenoids after véraison occurs [29-32]. This decline in the carotenoid content of grape berries appears to be associated with the disappearance of chloroplasts [30] and the formation of important aromatic C_13_-norisoprenoids (e.g. β-ionone, β-damascenone and vitispirane) in a number of cultivars including Sauvignon blanc [24,33]. Crupi et al. [34] suggested that this change in carotenoid concentration from véraison to harvest can be correlated with the ultimate flavour and aroma of grapes and wine. As alluded to by Kamffer et al. [35] and subsequently demonstrated by Lashbrooke et al. [36]; the analysis of the carotenoid and chlorophyll content of grapevine berries is technically quite challenging due to the susceptibility of these compounds to degradation. Papers utilising HPLC to determine carotenoid content often show evidence of chlorophyll degradation products in the respective chromatograms (i.e. pheophytin) (e.g. [32,35,37]). It is generally not reported if this is an artifact of the extraction method, or a true reflection of the carotenoid content of the berries [32,35]. Lashbrooke et al. [36], however, showed that conditions leading to the degradation of chlorophylls (and the concomitant formation of pheophytin) also lead to a degradation of carotenoids. Since degradation is not constant for all the pigments investigated (carotenoids and chlorophylls); an internal standard will not compensate accurately for this loss and this should be considered when attempting to correlate metabolite levels to, for example, transcript levels.

The total carotenoid concentration of the Sauvignon blanc berries showed a gradual decrease throughout the growth season with the lowest levels at harvest (Additional file 4). The concentration of only two carotenoids increased, namely: zeaxanthin and antheraxanthin (Additional file 4). In photosynthetic tissues, these two xanthophylls (together with violaxanthin) are involved in the xanthophyll (or violaxanthin) cycle: the reversible enzymatic conversion of violaxanthin to zeaxanthin (via the intermediate antheraxanthin). Under normal, non-stressed conditions, zeaxanthin is converted to violaxanthin by zeaxanthin epoxidase (ZEP). However, when photoprotection is required, violaxanthin is rapidly converted via antheraxanthin to zeaxanthin by violaxanthin de-epoxidase (VDE). Grapevine berries photosynthesise during the early stages of development in which case the carotenoids play a crucial role in the photosynthetic membranes by harvesting light and assisting in photoprotection [38-40]. The decline in total carotenoids closely follows the decline observed in total chlorophylls (carotenoid/chlorophyll ratios in Additional file 4). Only zeaxanthin and antheraxanthin showed an increase during ripening. Carotenoids have been shown to be more abundant in the skin versus the pulp or juice and although the abundance of carotenoids in berries is approximately 100-times lower than in leaves; the ratios of the photosynthetic pigments in berries are similar. Similar studies have shown comparable trends in carotenoid levels. In a water stress study conducted on berries of Cabernet Sauvignon and Chardonnay grapevine cultivars, the well-watered control plants of both cultivars showed a decrease in carotenoids during ripening [37]. Chlorophyll levels, however, were relatively low and antheraxanthin decreased and zeaxanthin could not be quantified at all. The authors attributed the low levels of chlorophylls (and the concomitant increase in pheophytin) as being artifacts of the extraction protocol, but added that carotenoid levels were unaffected. Lashbrooke et al. [36] demonstrated that carotenoids (especially the xanthophylls) are degraded under the conditions described in Deluc et al. [37] with the individual pigments having varying susceptibilities to degradation. Degradation would most likely have affected the concentration of other carotenoids, without necessarily affecting the observed trends.

Interestingly, the pigment analysis of the carotenoids showed the presence of lutein 5,6 epoxide; a xanthophyll involved in the lutein epoxide (Lx) cycle (reviewed in [41]) (Additional file 4 and Figure 2). The Lx cycle occurs in the α-carotene branch of the carotenoid biosynthetic pathway and functions similarly to the violaxanthin pathway (in the β-branch): during low light conditions (shade) there is an accumulation of lutein 5,6 epoxide that is de-epoxidised to lutein following light exposure. It is thought that the Lx cycle requires the same enzymes as the violaxanthin cycle (i.e. ZEP and VDE) [42], although there is some doubt whether lutein is a substrate for ZEP [43]. Kinetic experiments by Matsubara et al. [44] showed that the conversion of Lx to lutein is only slowly reversible. The authors speculated that the Lx cycle serves as an additional, slower reversible mechanism of photoprotection that supplements the violaxanthin cycle in shade plants. Not all plant species accumulate Lx, and the Lx cycle has been described as having an irregular taxonomical distribution in unrelated taxa [41]. Here we show that grapevine forms the carotenoids for both a violaxanthin cycle (violaxanthin and zeaxanthin) as well as an Lx cycle (lutein and lutein 5,6 epoxide); and possesses two isoforms for each of the required enzymes (i.e. ZEP and VDE) (Figure 2 and Additional file 4). Little is known about the regulation of these two pathways: that dedicated enzymes (ZEP and VDE) are required is improbable since the carotenoid substrates occur in the same localisation (the chloroplast), and whether or not transcriptional regulation is involved requires further study.

In later stages of berry development the products of the enzymatic cleavage of carotenoids (by the VvCCDs) are known to be potent flavour and aroma compounds (reviewed in [23]. It is possible that the CCDs have a recycling function converting their carotenoid substrates to volatile products that are desirable flavour and aroma compounds in grapes and wine. Interestingly the expression profiles of *VvCCD1.1* and *VvCCD1.2* increase up until véraison, and *VvCCD4a* and *VvCCD4b* increase dramatically throughout berry development and total carotenoid content concomitantly decreases (Figure 2 and Figure 3). Previous work in berries of Muscat of Alexandria and Shiraz [24], Trincadeira [45], and Chardonnay and Cabernet Sauvignon [31] grapevine cultivars showed that expression of the grapevine *VvCCD1* was induced approaching véraison. VvCCD1 was shown to be functional and can cleave both lutein and zeaxanthin, but not β-carotene, to form 3-hydroxy-β-ionone [24]. The authors showed a concomitant increase in the glycosylated and free forms of C_13_-norisoprenoids in berries of both cultivars (Muscat of Alexandria and Shiraz). It is currently not known if VvCCD1 is capable of cleaving additional carotenoids present in berries. VvCCD4a has recently been identified in grapevine and the authors reported the upregulation of the gene towards the end of ripening [27,28]. The authors suggest a possible role of CCD4a in berry colour, flavour and aroma of Chardonnay berries, even though functionality could not be demonstrated with a number of carotenoid substrates that included β-carotene and zeaxanthin [46]. Similarly, our data shows upregulation of *VvCCD4a* as well as *VvCCD4b* during ripening in Sauvignon blanc (Figure 2 and Additional file 3).

In grapevine, ABA, together with other phytohormones (e.g. ethylene), is thought to be responsible for the control of grape berry ripening and ABA levels typically peak at or around véraison [47]. Due to its importance, the components and regulation of the ABA biosynthetic pathway has similarly received much attention [48,49]. Wheeler et al. [50] showed that the expression pattern of two genes known to be crucial to ABA synthesis in plants (i.e. zeaxanthin epoxidase, *ZEP*; and a 9-*cis* epoxy carotenoid dioxygenase, *NCED*) could not be correlated to changes in ABA levels in grapevine berries. Sun et al. [47], however, showed that *VvNCED1* (AY337613) transcript levels correlated to the ABA content in peel, seed and pulp. These studies have illustrated the complex developmental regulation of ABA levels in a ripening berry [47,50,51]. Our results show that ABA levels increased from the initial green stage, peaking at véraison. A decrease in ABA occurs after véraison. It should also be noted that both skin and pulp (without seeds) were analysed collectively in this study. Grimplet et al. [52] have shown that more than 28% of transcripts in the berry display a more than 2-fold difference in transcript levels between the major tissue types: skin, pulp and seeds. The authors reported that most of the genes involved in carotenoid metabolism displayed a skin-specific expression pattern.

In a similar study, two VvNCED-encoding genes were identified in grapevine (*V. vinifera* L. cv. Shiraz): *VvNCED1* (Genbank accession number AY337613; *VvNCED3* in Additional file 1) and *VvNCED2* (Genbank accession number AY337614; *VvNCED2* in Additional file 1) [53]. The authors reported that the *VvNCED1* (*VvNCED3* in this study) and *VvNCED2* (*VvNCED2* in this study) cDNA sequences are 71.8% identical. Based on expression studies they showed that the majority of the detectable *NCED* expression in leaf tissue was *VvNCED1*-derived and that *VvNCED2* transcription was comparatively low and appears to be associated with the leaf ABA concentration. The authors hypothesised that VvNCED1 responds to stresses (such as water loss), whereas VvNCED2 performs more of a “house-keeping” function in leaves [53]. A study by Lund et al. [54] on Cabernet Sauvignon berries showed that *VvNCED1* expression was not significantly changed during berry ripening initiation (véraison), whereas *VvNCED2* showed upregulation in the later stages of ripening initiation. VvNCED2 was also shown to be upreguated in maturing seeds in the same study.

From the expression analysis in Sauvignon blanc grape berry from our study (Figure 2 and Figure 3) it is clear that the three *VvNCEDs* have distinctly different expression profiles. *VvNCED2* expression decreases as berry ripening progresses; whereas *VvNCED3* expression peaks at véraison. *VvNCED1* shows low expression levels in all of the berry stages, but increases after véraison. Since it has been demonstrated that the ABA levels peak at véraison (Additional file 5) [47], the transcriptomic analysis from this study supports the findings of Sun et al. [47] that *VvNCED3* is the enzymatic isoform correlated to the content of ABA in Sauvignon blanc berries.

The role and possible interplay of the NCED isoforms in the different tissue types of berries, as well as other grapevine tissues/organs, requires further elucidation. This study analysed *VvNCED* expression and ABA levels in the skin and pulp collectively without the seeds. Differential expression of *VvNCEDs* has previously been reported in the skin (exocarp), pulp (mesocarp) and seed of Cabernet Sauvignon during ripening initiation (véraison) with *VvNCED2* being transcriptionally upregulated at the later stages of véraison [54].

Due to the role of carotenoids and apocarotenoids to abiotic stress (e.g. the role of the xanthophylls in light stress; and ABA in water stress) and quality factors (e.g. the apocarotenoids and flavour and aroma formation); these compounds have been extensively studied (reviewed in [55] and references within). For example, the effect of environmental conditions on carotenoid metabolic genes is well known, but typically studied via a candidate gene approach. Collectively these studies have revealed the complex nature of carotenoid regulation that can occur at multiple levels, and although relatively well studied biochemically, the transcriptional regulation of this pathway is still not well understood. In general, it is becoming increasingly clear that an integrated analysis at the molecular level is required to elucidate function and gene-to-metabolite and/or metabolite-to-metabolite interactions.

Similarly, several of the isoforms of the carotenoid metabolic genes have distinctly different expression profiles and thus one can speculate that they have distinctly different functions in the developing grape berry and/or other grapevine organs. Further work investigating the environmental factors and relative changes in other plant metabolites present in berries at these stages will contribute to our understanding of the regulation of these genes.

From the cluster analysis in Figure3 it seems that a number of the carotenoid metabolic genes with similar profiles are possibly co-regulated/co-responding. Analysis of the upstream promoter sequences of these gene clusters could aid in the identification of berry-specific/developmental-specific transcription factor binding sites. Although not addressed in this study, the availability of two grapevine genomic sequence makes this approach feasible.

## **Conclusions**

Forty-two *Vitis vinifera* carotenoid metabolic pathway genes have been putatively identified and eleven have been isolated, sequenced and characterised in this study. The genes and their predicted protein sequences are highly conserved as reported in other plant studies. The grapevine genomic sequence facilitates the characterisation of gene functions and interactions, but more importantly facilitates the study of the complex frameworks between genes controlling metabolic pathways and ultimately the relationship of genes to phenotype. In a crop like grapevine, the ability to analyse the molecular “phenotype” is useful for quantifying the impact of, for example, a viticultural practice or a specific stress condition (biotic and/or abiotic).

The genes of the carotenoid metabolic pathway potentially form the basis for a number of genomic applications that include: gene-associated molecular marker developments (e.g. functional markers), biochemical characterisation of the corresponding recombinant proteins and transgenic approaches to manipulate carotenoid biosynthesis. Collectively these data form the most up-to-date pathway analysis and a baseline that will broaden our understanding of this central metabolic pathway and provides insights into the evolution of these compounds that also serve as substrates for quality impact factors (i.e. β-ionone, β-damascenone and vitispirane) and regulating phytohormones (i.e. ABA and strigolactone).

## **Methods**

### **Plant material**

*Vitis vinifera* L. cv. Pinotage leaf tissue and Sauvignon blanc berries were collected in the field and immediately flash frozen in liquid nitrogen. Berries were collected throughout the growth season (from the Stellenbosch and Elgin regions of South Africa from December through March) and flash frozen in the field in liquid nitrogen. The frozen tissue was ground in liquid nitrogen and, if not used immediately, stored at −80°C. At least three independent berry samples were collected (with n ≥ 50 berries per sample) at three time points: at pre-véraison/green stage (Eichhorn-Lorenz (E-L) system stage 31), at véraison (E-L stage 34) and at ripe/harvest stage (E-L stage 38) [56].

### **Plasmids, bacterial strains and growth conditions**

*Escherichia coli* cultures were grown in LB media, and transformed cultures were grown in LB media supplemented with the appropriate antibiotic(s) [57]. Unless otherwise stated, all bacterial cultures were grown at 37°C. The plasmids for the functional complementation assay (i.e. pAC-85b, pAC-LYC, pAC-BETA, and pAC-ZEAX) were obtained from F. X. Cunningham (Department of Cell Biology and Molecular Genetics, University of Maryland, MD, USA) and are described in [20,21].

### **Isolation and manipulation of nucleic acids**

All DNA fragments for cloning were separated in 1.0% (w/v) agarose TAE gels and the fragments of interest were isolated using the QIAquick Gel Extraction Kit as instructed by the supplier (Qiagen GmbH, Hilden, Germany). High molecular weight genomic DNA was isolated from fully expanded *V. vinifera* leaves as described in [58]. Total RNA from grapevine tissues was extracted according to the methods described in [59]. Unless otherwise stated, all standard methods for plasmid DNA isolation, manipulations and cloning of DNA fragments, and agarose gel electrophoresis were used as described in [57].

PCR reactions were performed using 10–50 ng of genomic DNA or cDNA as template. PCR amplifications were performed in an Applied Biosystems 2720 PCR thermal cycler (Applied Biosystems) using the following programme: an initial denaturation at 94°C for 5 min; subsequent denaturation at 94°C for 30 s; annealing at 5°C lower than the respective primer’s Tm for 30 s and extension at 72°C for 1 min per kbp (refer to Additional file 6 for the respective amplicon sizes and primer Tm) for 30 cycles; with a final elongation at 72°C for 10 min.

### **cDNA synthesis for full-length gene isolation**

cDNA was synthesised from 1 μg of DNase I-treated (Promega, Madison, WI) total RNA from Pinotage tissue samples using the sup III Platinum first strand synthesis system (Invitrogen) in a 20 μL reaction volume as described by the supplier. A duplicate reaction was performed without reverse transcriptase to verify the absence of genomic DNA in the RNA extractions (data not shown).

### **Construction of vectors**

Additional file 6 lists the PCR primers used to amplify the full-length carotenoid biosynthetic/catabolic genes from cDNA. The PCR-generated fragments were gel-purified and cloned into the pGEM-T Easy® vector system according to the specifications of the supplier (Promega) (Additional file 7 lists the constructed vectors). Cloned PCR-amplified products were sequenced with an ABI Prism 3100 Genetic Analyser at the Central Analytical Facility, Stellenbosch University, South Africa.

### **NimbleGen 12x135K arrays**

Global gene expression analysis was done from grape berries harvested at three time points with three repeats per time point: (1) green stage (E-L stage 31), (2) véraison (E-L stage 34) and (3) ripe/harvest stage (E-L stage 38), using the Roche NimbleGen Grape Whole-Genome array (Madison, WI in collaboration with Dr Massimo Delladonna from the Department of Biotechnology, University of Verona, Italy). The E-L stages were determined as described in [56]. Representative berries were sampled for all three stages. The arrays were run by a commercial enterprise, MoGene (St. Louis, MO), according to the instructions and recommendations of the supplier (NimbleGen). The resulting probe intensities were background corrected and normalised using a Robust Multichip Average (RMA) [60-62] and differentially expressed genes were identified/assessed using the Limma package (http://bioinf.wehi.edu.au/limma/) [63]. Data was deposited to the NCBI Gene Expression Omnibus (GEO) (accession number GSE34634).

### **Statistical analysis**

The microarray data was processed in R and background corrected and normalised with RMA ([61]. The Nimblegen probe sequences were mapped to the Grapevine Genome by the use of blastn [64]. Statistical significance of differential changes in gene expression, pigment concentrations and ABA concentrations between the green, véraison and ripe/harvest developmental stages was determined by the use of the Benjamini-Hochberg method [65] as implemented in Qlucore version 2.2 (Lund, Sweden) with a q-value threshold of 0.05.

### ***In silico*analyses**

The National Center for Biotechnology Information (NCBI) Entrez search and retrieval system was used to obtain nucleotide and protein sequences from the Genbank databases (http://www.ncbi.nlm.nih.gov/Entrez/). Alignments to sequences in the Genbank databases were performed using the relevant BLAST algorithm (http://www.ncbi.nlm.nih.gov/BLAST/) [64]. Nucleotide- sequences were aligned using ClustalX [66] and Dialign [67-69]. Amino acid sequences were aligned with MUSCLE [70]. Molecular phylogenetic analysis was performed with PhyML [17] and visualised using Treeview [71] and Figtree (http://tree.bio.ed.ac.uk/software/figtree/). Comparative genomic analyses (gene structure prediction, homologue/orthologue retrieval, and phylogenetic analyses) were performed via Plaza (http://bioinformatics.psb.ugent.be/plaza/) [4,5].

Intron- and exon splice sites in the genomic sequences were determined by aligning the cDNA and genomic sequences (Spidey: http://www.ncbi.nlm.nih.gov/spidey/) [72]. The putative sub-cellular localisation of protein sequences were predicted using ProtComp (http://www.softberry.com/berry.phtml). *V. vinifera* expressed sequence tags (ESTs) were retrieved from The Institute for Genomic Research (TIGR) Grape Gene Index (http://compbio.dfci.harvard.edu/tgi/) or NCBI. The *V. vinifera* genomic sequences were retrieved from NCBI, Genoscope (PN40042 genome sequence: http://www.cns.fr/externe/GenomeBrowser/Vitis/) and IASMA/FEM (ENTAV115 genome sequence: http://genomics.research.iasma.it).

### **Identification of putative carotenoid and apocarotenoid biosynthetic and catabolic genes**

*Arabidopsis thaliana* isoprenoid pathways and respective genes from AtIPD (http://www.atipd.ethz.ch/) [16] were used to identify putative *V. vinifera* orthologues via PLAZA (http://bioinformatics.psb.ugent.be/plaza/). Similarly, the annotations relating specifically to carotenoid biosynthesis were obtained from the VitisNet database (section 1.9: “Biosynthesis of Secondary Metabolites: Carotenoid Biosynthesis” http://www.sdstate.edu/aes/vitis/pathways.cfm) [73] and the corresponding accessions retrieved from PLAZA. The carotenoid and apocarotenoid biosynthetic and catabolic pathway used for Mapman [74] visualisation of expression and metabolite data was constructed using pathway information obtained from KEGG (map00906), AtIPD and VitisNet. Unique Roche Nimblegen probes were identified for the 42 accessions listed in Additional file 1. For simplicity, the nomenclature of the identified *V. vinifera* orthologues are based on sequence similarity to the closest *A. thaliana* orthologues, but numbered in ascending order relative to their chromosomal localization on the ENTAV115 genome sequence [1].

### **Positioning of the carotenoid metabolic genes in the grapevine genome**

The carotenoid metabolic genes listed in Additional file 1 were positioned on the genome sequence assembly described in [1]. The carotenoid biosynthetic/catabolic genes were mapped to the respective linkage groups of the heterozygous ENTAV115 genome sequence as position on the assembled linkage groups (in bp) as well as relative to representative markers of the genetic map described in [18].

### **Bacterial functional complementation: Pigment extraction and HPLC analysis**

*Escherichia coli* cultures containing the plasmids expressing carotenoid biosynthetic genes from *Erwinia herbicola* were used for the functional complementation assay as previously described in [20]. Briefly, for functional complementation of enzymes acting on lycopene an *E. coli* culture accumulating lycopene (via pAC-LYC), was transformed with a plasmid carrying the putative lycopene ε-cyclase encoding gene (*VvLECY1* from pGEM-cLECY1) or a plasmid carrying the putative lycopene β-cyclase encoding gene (*VvLBCY2* from pGEM-cLBCY2). For functional complementation of phytoene synthase (PSY) an *E. coli* culture accumulating phytoene (via pAC-85b), was transformed with a plasmid carrying the putative PSY (*VvPSY1* from pGEM-cPSY1). Similarly, a β-carotene accumulating strain (via pAC-BETA) was transformed with a plasmid carrying the putative β-carotene hydroxylase encoding gene (*VvBCH1* from pGEM-cBCH1). The functionality of the carotenoid genes was determined by analysing the pigment content of the cultures by reverse phase (RP)-HPLC. Care was taken to avoid light and air exposure to the cells during incubation and the subsequently isolated pigments. Pigments were extracted from 5 mL of an overnight culture by harvesting the cells by centrifugation (4,000 × g for 5 min at room temperature). The media was decanted and the cells were resuspended in sterile water in order to remove residual media components, and recovered by centrifugation (as above). The cells were vortexed briefly to loosen the pellet, and resuspended in 1 mL acetone. The extraction was placed at 65°C for 10 min with subsequent centrifugation at 13,000 × g for 10 min, and the supernatant containing the pigments was aspirated into a clean 2 mL microfuge tube. The extracted pigments were concentrated by centrifugation in a DNA110 Speed Vac concentrator (Savant Instruments, Inc., Farmingdale, NY). The recovered pigments were resuspended in ethyl acetate:methanol (1:4) and separated by RP-HPLC as described in [75].

### **Extraction and HPLC analysis of carotenoids and chlorophylls in grapevine berries**

Carotenoids and chlorophylls were extracted from grapevine berries and analysed by RP-HPLC as described in [36].

### **Extraction and UPLC-MS/MS analysis of abscisic acid**

Extraction of ABA from grapevine berries was adapted from Feurtado et al. [76]. Seeds were removed from berries, prior to freezing in liquid nitrogen and homogenisation in a bead mill. To extract the samples, 1 mL extraction solvent (80% isopropanol/1% acetic acid/19% water) and glass beads were added to 50 mg tissue. The samples were shaken at 200 rpm for 30 min at 4°C before centrifuging. Following collection of the supernatant, pellets were rinsed with 0.5 mL of extraction solvent. The combined supernatants were lyophilised, then reconstituted in 100 μL of acidified methanol and adjusted to 1 mL in acidified water. The reconstituted samples were passed through equilibrated 3 cm^3^ Oasis hydrophilic lipophilic balance (HLB) solid-phase extraction cartridges (Waters, Milford, MA, USA). After washing with 5% methanol, analytes were eluted with 80% acidified methanol water and lyophilised. Lyophilisates were dissolved in 200 μL 15% acetonitrile/0.07% acetic acid and clarified by centrifugation prior to transfer to UPLC-MS/MS analysis. ABA was quantified using a standard curve.

UPLC-MS/MS analyses were performed on a Waters Xevo triple quadrupole mass spectrometer coupled to a Waters Acquity UPLC. Separation was achieved on a Waters UPLC BEH Phenyl column (2.1x100 mm, 1.7 μm particle size). A 0.1% formic acid to acetonitrile gradient was used. The gradient was increased from 15% acetonitrile to 70% after 180 s and 95% after 190 s and returned to initial composition for a total run time of 5 min. The injection volume was 10 μL and a column temperature of 40°C was maintained. Solvents were LCMS grade and supplied by Sigma-Aldrich. Data acquisition was in multiple reaction monitoring mode (MRM). The precursor/product ions monitored were 263 > 153 and 263 > 219.2 (cone voltage 20 V, collision energy 10 V). The source temperature was 100°C, desolvation temperature was 400°C and desolvation gas of 600 L/h was applied, the remainder of the MS settings were optimised for the best possible sensitivity.

## **Competing interests**

The authors declare that they have no competing interests.

## **Authors' contributions**

MV and PY conceptualised the study. PY, EA, DJ and MV were involved in the experimental layout. PY, JL, EA and DJ did the field sampling. EA processed the samples for RNA isolation for subsequent microarray analysis. PY and JL processed the samples for pigment extractions and performed the HPLC analysis. PY, JL and DJ created the pathway visualisation. DJ provided statistical and bioinformatics support for the study. PY isolated and cloned the genes of interest and performed the bacterial functional complementation assay. CM and RV performed the mapping of the identified genes to the respective linkage groups. PY and MV drafted the initial manuscript. All authors contributed to discussion of the results, reviewing of the manuscript and approved the final manuscript.

## Supplementary Material

Additional file 1 **Gene names, relevant accession numbers and putative gene assignments for the predicted genes encodingcarotenoid biosynthetic and catabolic enzymes.** Gene sequences isolated in this study are underlined. Click here for file

Additional file 2 **Chromosomal localisation of the carotenoid metabolic genes.** The 37 of the 42 carotenoid metabolic pathway members are depicted on the heterozygous ENTAV115 *V. vinifera* L. cv Pinot noir genome sequence assembly together with the closest genetic markers and with other well distributed markers along the chromosomes as taken from Troggio et al. [[18].]. The genes from Additional file 2: Table SM1 are in italic (red); isolated genes are in bold italic (red). Relative positions on each chromosome in bp are indicated on the left of each linkage group (LG). Click here for file

Additional file 3 **Expression of the carotenoid biosynthetic/catabolic genes at the three berry developmental stages.** Average expression values of the carotenoid metabolic genes at the three stages of berry development (E-L stage 31, -34 and −38). Average expression values from the Nimblegen whole-genome grape arrays are shown with their standard deviations (n = 3). Genes in bold indicate significant differential expression (q-value ≤ 0.05; n = 3) in the green stage (E-L stage 31) versus véraison stage (E-L stage 34)^a^; véraison stage (E-L stage 34) versus ripe/harvest stage (E-L stage 38)^b^; green stage (E-L stage 31) versus ripe/harvest stage (E-L stage 38)^c^. Click here for file

Additional file 4 **Photosynthetic pigments concentrations and ratios in three berry developmental stages.** Photosynthetic pigments extracted from green, véraison and ripe/harvest stage berries were separated by HPLC and quantified relative to authentic standards. Average carotenoid and chlorophyll concentrations in berries are shown in ng/mg FW, with the respective standard deviations (n = 3). Pigments in bold indicate significant differences (q-value ≤ 0.05; n = 3) in pigment concentrations in the green stage (E-L stage 31) versus véraison stage (E-L stage 34)^a^; véraison stage (E-L stage 34) versus ripe/harvest stage (E-L stage 38)^b^; green stage (E-L stage 31) versus ripe/harvest stage (E-L stage 38)^c^. Click here for file

Additional file 5 **Abscisic acid concentration in the three berry developmental stages.** Abscisic acid was extracted from the three stages of berry development (E-L stage 31, -34 and −38) and analysed using UPLC MS/MS and quantified relative to an authentic standard. Abscisic acid concentrations in berries are shown in ng/g FW, with the respective standard deviations (n = 3). Significant differences in ABA concentrations (q-value ≤ 0.05; n = 3) in the green stage (E-L stage 31) versus véraison stage (E-L stage 34)^a^; véraison stage (E-L stage 34) versus ripe/harvest stage (E-L stage 38)^b^; green stage (E-L stage 31) versus ripe/harvest stage (E-L stage 38)^c^. Click here for file

Additional file 6 **PCR primers used in this study.** The table lists the primers used, the respective sequences, melting temperatures (Tm’s) and a brief description of the amplified product. Where applicable, restriction sites incorporated to facilitate cloning are indicated in lowercase letters in the respective primer sequence. Click here for file

Additional file 7 **Plasmids and constructs used in this study.** Plasmids constructed in this study were named according to the carotenoid biosynthetic/catabolic gene they contained. The primers used and the size of the PCR product cloned are listed in the respective columns. Click here for file
