# Supplementary material for: The genes and enzymes of the carotenoid metabolic pathway in Vitis vinifera L
Source: BMC Genomics. 2012 Jun 15;13:243. doi: 10.1186/1471-2164-13-243 (PMC3484060; doi:10.1186/1471-2164-13-243)
Supplement: Additional file 1 — Gene names, relevant accession numbers and putative gene assignments for the predicted genes encodingcarotenoid biosynthetic and catabolic enzymes. Gene sequences isolated in this study are underlined. [file 1471-2164-13-243-S1.doc]

| **Gene name** | **PLAZA 1.0**  **accession^1^** | **PLAZA 2.0**  **accession^2^** | **Roche Nimblegen**  **Probe accession^3^** | ***Arabidopsis* *thaliana***  **orthologue accession^4^** | **Putative gene assignment** |
| --- | --- | --- | --- | --- | --- |
| *VvPSY1* | VV00G37410 | VV04G07200 | CHR4_JGVV79_53_T01 | At5g17230 | phytoene synthase (PSY) |
| *VvPSY2* | VV12G00970 | VV12G03800 | CHR12_JGVV28_288_T01 | At5g17230 | phytoene synthase (PSY) |
| *VvPDS1* | VV09G00040 | VV09G01520 | CHR9_JGVV2_7_T01 | At4g14210 | phytoene desaturase (PDS) |
| *VvPDH2* | VV04G11160 | VV04G02170 | CHR4_JGVV23_199_T01 | n/a | phytoene dehydrogenase-related (PDH) |
| *VvPDH1* | VV04G11150 | VV04G02180 | CHR4_JGVV23_200_T01 | n/a | phytoene dehydrogenase-related (PDH) |
| *VvZISO1* | VV05G13300 | VV05G10630 | CHR5_JGVV62_90_T01 | At1g10830 | 15-*cis*-ζ-carotene isomerase (Z-ISO) |
| *VvZDS1* | VV14G05860 | VV14G03510 | CHR14_JGVV30_54_T01 | At3g04870 | ζ-carotene desaturase (ZDS) |
| *VvCISO1* | VV08G02490 | VV08G01150 | CHR8_JGVV32_65_T01 | At1g06820 | carotenoid isomerase (CISO2) |
| *VvCISO2* | VV00G42740 | VV12G07010 | CHR12_JGVV35_105_T01 | At1g57770 | carotenoid isomerase (CISO1) |
| *VvLBCY1* | VV00G44920 | VV08G09850 | CHR6_JGVV80_42_T01 | At3g10230 | lycopene β-cyclase (LBCY2) |
| *VvLBCY2* | VV08G15130 | VV08G09850 | CHR8_JGVV7_313_T01 | At3g10230 | lycopene β-cyclase (LBCY1) |
| *VvLECY1* | VV11G01840 | VV11G03280 | CHR11_JGVV16_157_T01 | At5g57030 | lycopene epsilon-cyclase (LECY) |
| *VvLUT1* | VV08G13950 | VV08G10880 | CHR8_JGVV7_421_T01 | At3g53130 | carotene hydroxylase (CYP97C1; LUT1) |
| *VvLUT5* | VV04G09060 | VV04G03730 | CHR4_JGVV23_337_T01 | At1g31800 | carotene hydroxylase (CYP97A3; LUT5) |
| *VvBCH1* | VV02G00220 | VV02G03540 | CHR2_JGVV25_22_T01 | At4g25700 | β-carotene hydroxylase (BCH1) |
| *VvBCH2* | VV00G45980 | VV16G07970 | CHR16_JGVV50_151_T01 | At5g52570 | β-carotene hydroxylase (BCH2) |
| *VvZEP1* | VV07G11310 | VV07G05510 | CHR7_JGVV31_55_T01 | At5g67030 | zeaxanthin epoxidase (ZEP; ABA1) |
| *VvZEP2* | VV00G57140 | VV13G00730 | CHR13_JGVV156_33_T01 | At5g67030 | zeaxanthin epoxidase (ZEP; ABA1) |
| *VvVDE1* | VV00G14320 | VV04G04430 | CHR4_JGVV43_68_T01 | At1g08550 | violaxanthin de-epoxidase (VDE1) |
| *VvVDE2* | VV07G12510 | VV07G06540 | CHR7_JGVV31_158_T01 | At2g21860 | violaxanthin de-epoxidase (VDE2) |
| *VvNSY1* | VV14G07860 | VV14G08050 | CHR14_JGVV6_184_T01 | At1g67080 | neoxanthin synthase (NSY) |
| *VvNCED1* | VV05G10260 | VV05G09670 | CHR5_JGVV51_51_T01 | At3g24220 | 11,12 9-*cis* epoxycarotenoid dioxygenase (NCED1) |
| *VvNCED2* | VV10G02580 | VV10G04440 | CHR10_JGVV3_335_T01 | At3g14440 | 11,12 9-*cis* epoxycarotenoid dioxygenase (NCED2) |
| *VvNCED3* | VV19G09570 | VV19G11960 | CHR19_JGVV93_38_T01 | At1g78390 | 11,12 9-*cis* epoxycarotenoid dioxygenase (NCED3) |
| *VvABA2* | VV00G44805 | VV06G09640 | CHR6_JGVV80_22_T01 | At1g52340 | short-chain alcohol dehydrogenase (ABA2) |
| *VvAAO3* | VV18G17270 | VV18G13270 | CHR18_JGVV41_5_T01 | At2g27150 | abscisic aldehyde oxidase (AAO3) |
| *VvA8H-CYP707A1* | VV02G12060 | VV02G09270 | CHR2_JGVV87_27_T01 | At4g19230 | Abscisic acid 8` hydroxylase (CYP707A1) |
| *VvA8H-CYP707A2.1* | VV06G05860 | VV06G03270 | CHR6_JGVV4_319_T01 | At2g29090 | Abscisic acid 8` hydroxylase (CYP707A2) |
| *VvA8H-CYP707A2.2* | VV07G11390 | VV07G05570 | n/a | At2g29090 | Abscisic acid 8` hydroxylase (CYP707A2) |
| *VvA8H-CYP707A2.3* | VV18G08790 | VV18G07120 | CHR18_JGVV1_715_T01 | At2g29090 | Abscisic acid 8` hydroxylase (CYP707A2) |
| *VvA8H-CYP707A2.4* | VV18G17300 | VV18G13280 | n/a | At2g29090 | Abscisic acid 8` hydroxylase (CYP707A2) |
| *VvA8H-CYP707A2.5* | VV18G17320 | VV18G13300 | CHR18_JGVV41_8_T01 | At2g29090 | Abscisic acid 8` hydroxylase (CYP707A2) |
| *VvA8H-CYP707A2.6* | VV00G05785 | VV06G12620 | CHR6_JGVV9_252_T01 | At2g29090 | Abscisic acid 8` hydroxylase (CYP707A2) |
| *VvA8H-CYP707A4* | VV03G06860 | VV03G06660 | CHR3_JGVV63_36_T01 | At3g19270 | Abscisic acid 8` hydroxylase (CYP707A4) |
| *VvCCD4a* | VV02G12270 | VV02G09110 | CHR2_JGVV87_11_T01 | At4g19170 | (9,10) (9`,10`) cleavage dioxygenase (CCD4) |
| *VvCCD4b* | VV02G12290 | VV02G09110 | CHR2_JGVV87_9_T01 | At4g19170 | (9,10) (9`,10`) cleavage dioxygenase (CCD4) |
| *VvCCD4c* | VV16G01030 | VV16G05200 | CHR16_JGVV39_78_T01 | At4g19170 | (9,10) (9`,10`) cleavage dioxygenase (CCD4) |
| *VvCCD1.1* | VV13G12460 | VV13G07870 | CHR13_JGVV64_95_T01 | At3g63520 | (5,6) (5`,6`) (9,10) (9`,10`) cleavage dioxygenase (CCD1) |
| *VvCCD1.2* | VV13G12530 | VV13G07840 | CHR13_JGVV64_94_T01 | At3g63520 | (5,6) (5`,6`) (9,10) (9`,10`) cleavage dioxygenase (CCD1) |
| *VvCCD7* | VV15G01930 | VV15G00750 | CHR15_JGVV21_57_T01 | At2g44990 | (9`,10`) cleavage dioxygenase (CCD7; MAX3) |
| *VvCCD8* | VV04G03390 | VV04G10240 | CHR4_JGVV8_318_T01 | At4g32810 | (13,14) cleavage dioxygenase (CCD8; MAX4) |
| *VvMAX1* | VV04G01070 | VV04G08200 | CHR4_JGVV8_99_T01 | At2g26170 | cytochrome P450 (CYP711A1; MAX1) |

^1^ PLAZA version 1: <http://bioinformatics.psb.ugent.be/plaza_v1/>

^2^ PLAZA version 2: <http://bioinformatics.psb.ugent.be/plaza/>

^3^ Probe accession for the custom Nimblegen grape arrays (12 x 135K)

^4^ Probe accession for the *Arabidopsis thaliana* orthologue
