# Supplementary figures and images for: The genes and enzymes of the carotenoid metabolic pathway in Vitis vinifera L
Source: BMC Genomics. 2012 Jun 15;13:243. doi: 10.1186/1471-2164-13-243 (PMC3484060; doi:10.1186/1471-2164-13-243)

## LG2

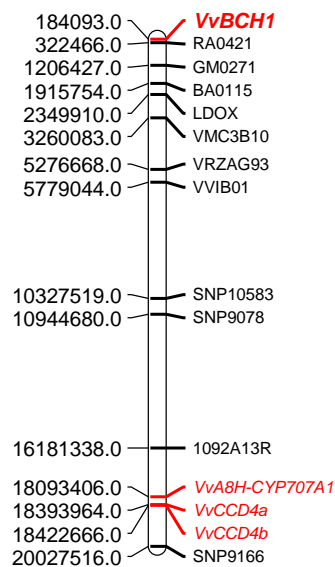

## LG3

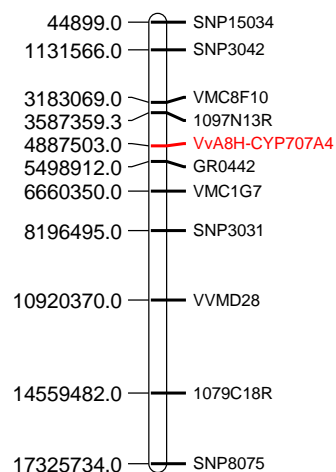

## LG4

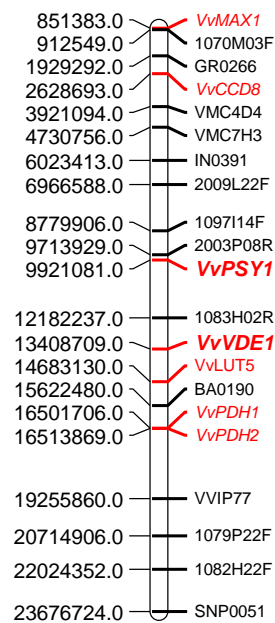

## LG6

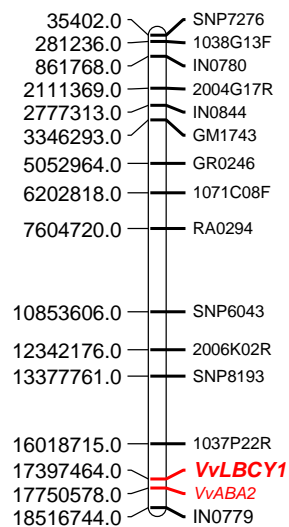

## LG7

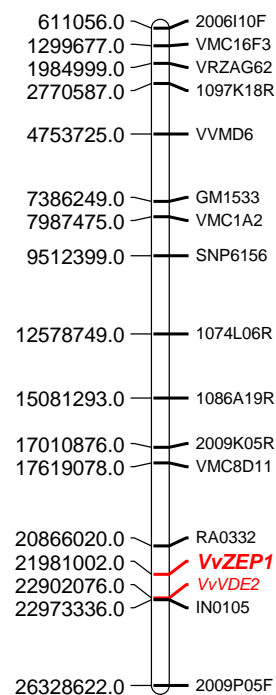

## LG8

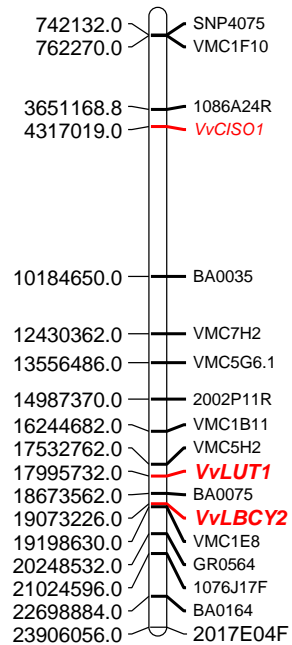

## LG11

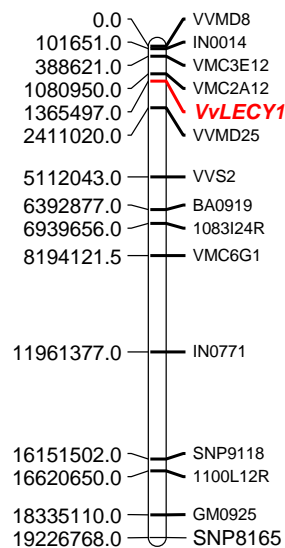

## LG12

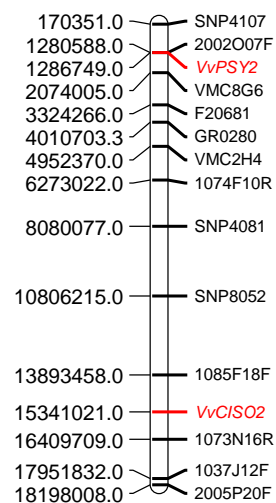

## LG13

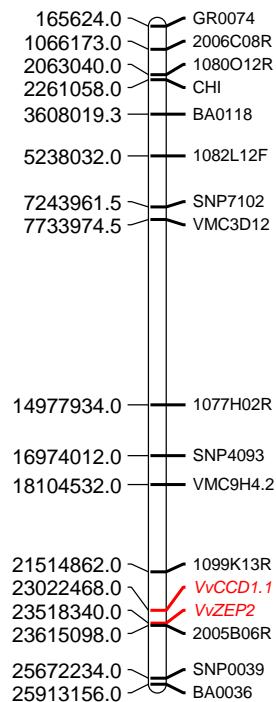

## LG16

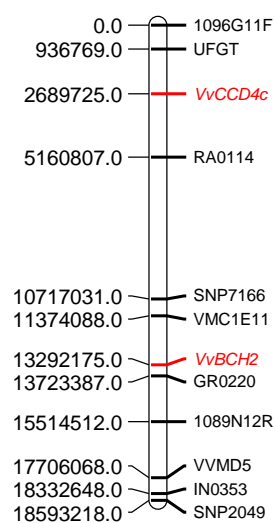

## LG18

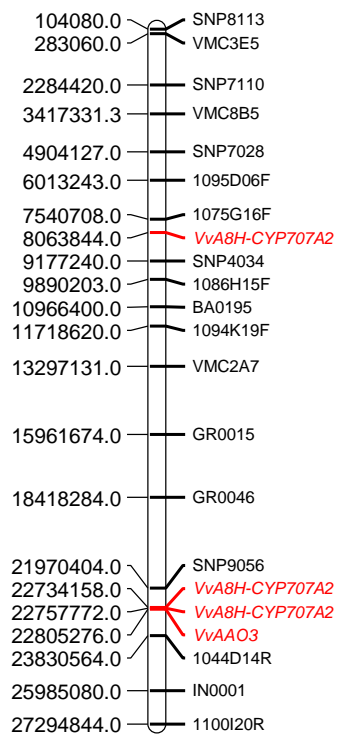

## LG19

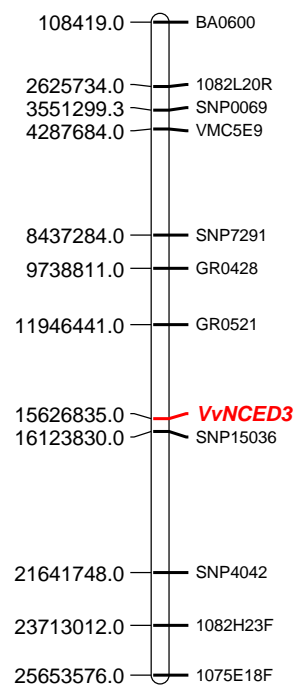

Supplement: Additional file 2 — Chromosomal localisation of the carotenoid metabolic genes. The 37 of the 42 carotenoid metabolic pathway members are depicted on the heterozygous ENTAV115 V. vinifera L. cv Pinot noir genome sequence assembly together with the closest genetic markers and with other well distributed markers along the chromosomes as taken from Troggio et al. [[18].]. The genes from Additional file 2: Table SM1 are in italic (red); isolated genes are in bold italic (red). Relative positions on each chromosome in bp are indicated on the left of each linkage group (LG). [file 1471-2164-13-243-S2.pdf]

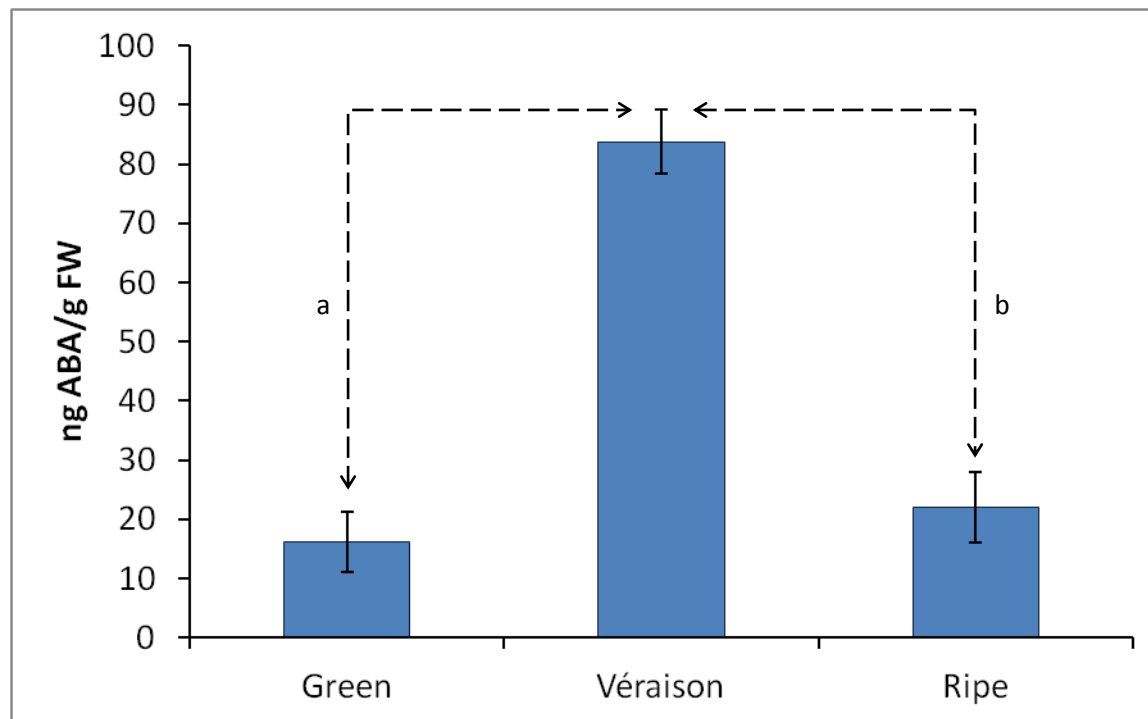

Supplement: Additional file 5 — Abscisic acid concentration in the three berry developmental stages. Abscisic acid was extracted from the three stages of berry development (E-L stage 31, -34 and −38) and analysed using UPLC MS/MS and quantified relative to an authentic standard. Abscisic acid concentrations in berries are shown in ng/g FW, with the respective standard deviations (n = 3). Significant differences in ABA concentrations (q-value ≤ 0.05; n = 3) in the green stage (E-L stage 31) versus véraison stage (E-L stage 34)a; véraison stage (E-L stage 34) versus ripe/harvest stage (E-L stage 38)b; green stage (E-L stage 31) versus ripe/harvest stage (E-L stage 38)c. [file 1471-2164-13-243-S5.pdf]
