# Supplementary material for: The genes and enzymes of the carotenoid metabolic pathway in Vitis vinifera L
Source: BMC Genomics. 2012 Jun 15;13:243. doi: 10.1186/1471-2164-13-243 (PMC3484060; doi:10.1186/1471-2164-13-243)
Supplement: Additional file 3 — Expression of the carotenoid biosynthetic/catabolic genes at the three berry developmental stages. Average expression values of the carotenoid metabolic genes at the three stages of berry development (E-L stage 31, -34 and −38). Average expression values from the Nimblegen whole-genome grape arrays are shown with their standard deviations (n = 3). Genes in bold indicate significant differential expression (q-value ≤ 0.05; n = 3) in the green stage (E-L stage 31) versus véraison stage (E-L stage 34)a; véraison stage (E-L stage 34) versus ripe/harvest stage (E-L stage 38)b; green stage (E-L stage 31) versus ripe/harvest stage (E-L stage 38)c. [file 1471-2164-13-243-S3.doc]

|  | **Green** | **Véraison** | **Ripe** |
| --- | --- | --- | --- |
| **Gene name** | **E-L stage 31** | **E-L stage 34** | **E-L 38** |
| ***VvNCED2***^c^ | 1788.37±224.44 | 1256.94±511.60 | 270.14±44.87 |
| ***VvLECY1***^abc^ | 4390.93±90.06 | 855.68±108.21 | 1984.36±314.32 |
| **VvPSY2**^abc^ | 1033.72±27.08 | 3221.74±222.71 | 1736.81±211.40 |
| ***VvCISO2***^c^ | 659.98±11.74 | 924.16±154.39 | 915.97±44.27 |
| ***VvZEP2***^ac^ | 2111.90±48.21 | 1113.69±258.69 | 1026.67±28.55 |
| ***VvCCD1.2***^a^ | 3411.74±9.05 | 7948.65±1376.65 | 5265.79±895.36 |
| ***VvCCD1.1***^c^ | 6881.14±312.18 | 9414.01±4464.89 | 2898.02±901.14 |
| ***VvZDS1***^ab^ | 7467.94±367.50 | 25221.38±407.30 | 9980.92±1776.81 |
| ***VvNSY1***^ab^ | 2046.41±152.19 | 5700.19±612.57 | 1480.59±231.75 |
| *VvCCD7* | 44.97±7.81 | 35.50±4.95 | 35.90±3.27 |
| *VvCCD4c* | 37.13±5.25 | 36.43±4.44 | 42.94±5.08 |
| ***VvBCH2***^ac^ | 3909.32±307.70 | 1179.23±234.49 | 1252.26±382.38 |
| ***VvA8H-CYP707A2.3***^ac^ | 4308.46±989.28 | 91.*96*±11.01 | 70.79±11.34 |
| ***VvAAO3***^c^ | 448.96±49.30 | 462.09±124.16 | 280.14±22.87 |
| *VvA8H-CYP707A2.5* | 203.21±30.52 | 283.94±13.90 | 211.87±34.85 |
| *VvNCED3* | 541.26±43.30 | 1127.36±344.02 | 489.79±97.18 |
| *VvBCH1* | 44.48±5.13 | 53.28±14.04 | 46.45±6.42 |
| *VvCCD4a* | 172.07±27.94 | 389.66±110.15 | 1470.08±600.85 |
| ***VvA8H-CYP707A1***^a^ | 739.91±154.49 | 1273.96±122.59 | 866.30±91.68 |
| ***VvCCD4b***^ac^ | 42.78±1.98 | 10635.04±1153.27 | 10377.82±2530.98 |
| *VvA8H-CYP707A4* | 53.06±4.84 | 47.56±4.45 | 76.64±28.36 |
| ***VvPDH2***^abc^ | 299.50±36.85 | 608.69±37.64 | 1511.31±209.39 |
| ***VvPDH1***^c^ | 3456.41±55.68 | 2748.83±353.51 | 1829.35±25.96 |
| *VvLUT5* | 7855.01±471.65 | 8205.62±305.50 | 7034.56±360.53 |
| *VvVDE1* | 657.95±10.29 | 777.88±177.49 | 702.94±26.37 |
| ***VvPSY1***^abc^ | 17670.95±312.44 | 5021.36±369.73 | 3014.92±124.81 |
| *VvCCD8* | 37.00±4.99 | 39.77±3.63 | 67.91±30.00 |
| ***VvMAX1***^a^ | 205.36±44.55 | 74.35±14.54 | 108.87±6.74 |
| ***VvNCED1***^abc^ | 40.25±4.45 | 71.96±4.81 | 112.80±11.40 |
| ***VvZISO1***^abc^ | 2566.35±135.67 | 5154.27±561.79 | 7558.69±472.60 |
| *VvA8H-CYP707A2.1* | 45.05±3.87 | 43.06±0.89 | 50.17±13.65 |
| *VvABA2* | 44.64±4.86 | 47.59±3.47 | 43.51±0.78 |
| ***VvLBCY1***^ac^ | 3020.44±195.99 | 1601.97±212.78 | 1078.10±84.80 |
| *VvA8H-CYP707A2.6* | 716.64±60.25 | 882.46±106.39 | 972.01±151.07 |
| ***VvVDE2***^c^ | 509.05±22.09 | 436.77±127.60 | 1165.33±257.93 |
| ***VvZEP1***^c^ | 6345.02±423.03 | 4537.32±675.84 | 3215.92±67.03 |
| ***VvCISO1***^ab^ | 6323.03±338.81 | 11277.61±920.90 | 6397.45±769.55 |
| ***VvLBCY2***^ac^ | 1694.87±74.82 | 2829.92±285.26 | 2818.45±254.97 |
| ***VvLUT1***^abc^ | 2504.28±73.92 | 1494.99±113.06 | 641.07±27.59 |
| ***VvPDS1***^c^ | 1796.36±124.76 | 1744.43±279.48 | 1224.56±6.76 |

^a^ Green stage (E-L stage 31) versus véraison stage (E-L stage 34);

^b^ Véraison stage (E-L stage 34) versus ripe stage (E-L stage 38); and

^c^ Green stage (E-L stage 31) versus ripe stage (E-L stage 38).
