# Supplementary material for: The genes and enzymes of the carotenoid metabolic pathway in Vitis vinifera L
Source: BMC Genomics. 2012 Jun 15;13:243. doi: 10.1186/1471-2164-13-243 (PMC3484060; doi:10.1186/1471-2164-13-243)
Supplement: Additional file 4 — Photosynthetic pigments concentrations and ratios in three berry developmental stages. Photosynthetic pigments extracted from green, véraison and ripe/harvest stage berries were separated by HPLC and quantified relative to authentic standards. Average carotenoid and chlorophyll concentrations in berries are shown in ng/mg FW, with the respective standard deviations (n = 3). Pigments in bold indicate significant differences (q-value ≤ 0.05; n = 3) in pigment concentrations in the green stage (E-L stage 31) versus véraison stage (E-L stage 34)a; véraison stage (E-L stage 34) versus ripe/harvest stage (E-L stage 38)b; green stage (E-L stage 31) versus ripe/harvest stage (E-L stage 38)c. [file 1471-2164-13-243-S4.doc]

|  | **Green** | **Véraison** | **Ripe** |
| --- | --- | --- | --- |
|  | **E-L stage 31** | **E-L stage 34** | **E-L 38** |
| **Violaxanthin** ^a,b,c^ | 0.98±0.09 | 0.69±0.06 | 0.36±0.06 |
| **Neoxanthin** ^a,b,c^ | 1.69±0.14 | 0.96±0.02 | 0.30±0.08 |
| **Lutein 5,6 epoxide** ^a,b,c^ | 1.07±0.11 | 0.36±0.05 | 0.04±0.08 |
| Antheraxanthin | 0.26±0.23 | 0.42±0.04 | 0.35±0.11 |
| **Lutein** ^a,b,c^ | 3.40±0.27 | 2.13±0.07 | 0.97±0.23 |
| **Zeaxanthin** ^c^ | 0.00±0.00 | 0.11±0.10 | 0.29±0.07 |
| **β-carotene** ^a,b,c^ | 1.83±0.09 | 1.17±0.01 | 0.49±0.05 |
| **Chlorophyll a** ^a,b,c^ | 29.16±1.58 | 18.30±0.09 | 7.08±1.11 |
| **Chlorophyll b** ^a,b,c^ | 17.01±1.60 | 10.51±0.52 | 4.08±0.46 |
| **Pheophytin a** ^a^ | 3.68±1.37 | 0.49±0.43 | 2.72±4.71 |
| Total carotenoids | 9.25 | 5.85 | 2.81 |
| Total Chlorophyll^1^ | 46.18 | 28.81 | 11.15 |
| Chlorophyll a/Chlorophyll b | 1.71 | 1.74 | 1.74 |
| Carotenoid/chlorophyll ratio | 0.20 | 0.20 | 0.25 |

^1^Total chlorophylls representing chlorophyll a and b, but excludes pheophytin a and b

^a^ Green stage (E-L stage 31) versus véraison stage (E-L stage 34);

^b^ Véraison stage (E-L stage 34) versus ripe stage (E-L stage 38); and

^c^ Green stage (E-L stage 31) versus ripe stage (E-L stage 38).
