# Supplementary material for: The genes and enzymes of the carotenoid metabolic pathway in Vitis vinifera L
Source: BMC Genomics. 2012 Jun 15;13:243. doi: 10.1186/1471-2164-13-243 (PMC3484060; doi:10.1186/1471-2164-13-243)
Supplement: Additional file 6 — PCR primers used in this study. The table lists the primers used, the respective sequences, melting temperatures (Tm’s) and a brief description of the amplified product. Where applicable, restriction sites incorporated to facilitate cloning are indicated in lowercase letters in the respective primer sequence. [file 1471-2164-13-243-S6.doc]

| **PRIMER** | **SEQUENCE (5’-3’)** | **Tm (°C)** | **PRODUCT DESCRIPTION** |
| --- | --- | --- | --- |
| VvPSY1_5’-ATG | ATGTCTGTTGCTCTGTTGTGGATTG | 60 | Amplifies the full-length phytoene synthase 1 encoding gene (*VvPSY1)* from cDNA (1317 bp) |
| VvPSY1_3’-STOP | CTCGAGGATGTCCATTCATGCCTTGACT | 65 |
| VvPDS1_5’-ATG | ctcgagATGACTCAATTCAGATATGTTTCTGTGG | 62 | Amplifies the full-length phytoene desaturase 1 encoding gene (*VvPDS1)* from cDNA (1749 bp) |
| VvPDS1_3’-STOP | TTAACTGAGAATGCTGACCTCGG | 60 |
| VvZDS1_5’-ATG | ATGTCTTCTTCTATTCTTTTTCCTGCT | 56 | Amplifies the full-length ζ-carotene desaturase 1 encoding gene (*VvZDS1)* from cDNA (1752 bp) |
| VvZDS1_3’-STOP | CCCGGGGGTGTGAGGTGTCAGACAAGACTC | 74 |
| VvLECY1_5’-ATG | gtcgacTCGTCTACATGGAGTGTCTCGGA | 68 | Amplifies the full-length lycopene ε-cylcase 1 encoding gene (*VvLECY1)* from cDNA (1593 bp) |
| VvLECY1_3’-STOP | CCAGGCTCCTCATCTAGTCTACTGC | 64 |
| VvLUT1_5’-ATG | ctcgagATGTCTCTCTCTTCTTTCGCACTCC | 66 | Amplifies the full-length ε-carotene hydroxylase 1 encoding gene (*VvECH1*) from cDNA (1662 bp) |
| VvLUT1_3’-STOP | TTACCTAGAAGATGTTGGAACAAATGC | 57 |
| VvLBCY2_5’-ATG | ATGGATACTTTACTCAAGACTCATAATAAGC | 56 | Amplifies the full-length lycopene β-cylcase 2 encoding gene (*VvLBCY2)* from cDNA (1515 bp) |
| VvLBCY2_3’-STOP | GTTCCATCATCTTAATCCTTGTCCTG | 59 |
| VvBCH1_5’-ATG | attaatATGGCGACAGGAATTTC | 53 | Amplifies the full-length β-carotene hydroxylase 1 encoding gene (*VvBCH1)* from cDNA (900 bp) |
| VvBCH1_3’-STOP | ATCGCTCGAGTGCATACTGATTGATGTCATATTC | 62 |
| VvZEP1_5’-ATG | gtcgacATGGCTTCAGCAGTGTTTTATAG | 61 | Amplifies the full-length zeaxanthin epoxidase 1 encoding gene (*VvZEP1)* from cDNA (1977 bp) |
| VvZEP1_3’-STOP | TCAAACCGCCTGGAAGAGCT | 62 |
| VvVDE1_5’-ATG | ATGGCATTGGCAGCACATC | 58 | Amplifies the full-length violaxanthin de-epoxidase 1 encoding gene (*VvVDE1)* gene from cDNA (1440 bp) |
| VvVDE1_3’-STOP | CAGGAATTAAACTGGGGTTTCAGG | 60 |
| VvLBCY1_5’-ATG | ctcgagATGGGAACGCTACTCTGG | 65 | Amplifies the full-length lycopene β-cylcase 1 encoding gene (*VvLBCY1)* gene from cDNA (1494 bp) |
| VvLBCY1_3’-STOP | TCAAATAGTTTCGAATGCCA | 52 |
| VvNCED3_5’-ATG | ATGGCTTCTCCTGCAGCTGC | 63 | Amplifies the full-length 9-*cis* epoxy carotenoid dioxygenase 3 encoding gene (*VvNCED3)* gene from cDNA (1833 bp) |
| VvNCED3_3’-STOP | CAATCTGACACCAAGCAGCCATG | 62 |
| Oligo-dT_16_ | TTTTTTTTTTTTTTTTN | 37 | Generic primer that binds to poly-A tail of mRNA, and primes the first-strand synthesis for reverse transcription/cDNA synthesis. |
