# Supplementary material for: The genes and enzymes of the carotenoid metabolic pathway in Vitis vinifera L
Source: BMC Genomics. 2012 Jun 15;13:243. doi: 10.1186/1471-2164-13-243 (PMC3484060; doi:10.1186/1471-2164-13-243)
Supplement: Additional file 7 — Plasmids and constructs used in this study. Plasmids constructed in this study were named according to the carotenoid biosynthetic/catabolic gene they contained. The primers used and the size of the PCR product cloned are listed in the respective columns. [file 1471-2164-13-243-S7.doc]

| **CONSTRUCT** | **DESCRIPTION** |
| --- | --- |
| pGEM-cPSY1 | The 1317 bp full-length *VvPSY1* was PCR-amplified from cDNA (using the primers VvPSY5’-ATG and VvPSY3’-STOP) and cloned into pGEM®-T Easy |
| pGEM-cPDS1 | The 1749 bp full-length Vv*PDS1* was PCR-amplified from cDNA (using the primers VvPDS5’-ATG and VvPDS3’-STOP) and cloned into pGEM®-T Easy |
| pGEM-cZDS1 | The 1752 bp full-length *VvZDS1* was PCR-amplified from cDNA (using the primers VvZDS5’-ATG and VvZDS3’-STOP) and cloned into pGEM®-T Easy |
| pGEM-cLECY1 | The 1593 bp full-length *VvLECY1* was PCR-amplified from cDNA (using the primers VvLECY5’-ATG and VvLECY3’-STOP) and cloned into pGEM®-T Easy |
| pGEM-cLUT1 | The 1692 bp full-length *VvECH1* was PCR-amplified from cDNA (using the primers VvECH5’-ATG and VvECH3’-STOP) and cloned into pGEM®-T Easy |
| pGEM-cLBCY1 | The 1494 bp full-length *VvLBCY1* was PCR-amplified from cDNA (using the primers VvLBCY15’-ATG and VvLBCY13’-STOP) and cloned into pGEM®-T Easy |
| pGEM-cLBCY2 | The full-length 1515 bp *VvLBCY2* was PCR-amplified from cDNA (using the primers VvLBCY25’-ATG and VvLBCY23’-STOP) and cloned into pGEM®-T Easy |
| pGEM-cBCH1 | The 900 bp full-length *VvBCH1* was PCR-amplified from cDNA (using the primers VvBCH5’-ATG and VvBCH3’-STOP) and cloned into pGEM®-T Easy |
| pGEM-cZEP1 | The 1977 bp full-length *VvZEP1* was PCR-amplified from cDNA (using the primers VvZEP5’-ATG and VvZEP3’-STOP) and cloned into pGEM®-T Easy |
| pGEM-cVDE1 | The 1440 bp full-length *VvVDE1* was PCR-amplified from cDNA (using the primers VvVDE5’-ATG and VvVDE3’-STOP) and cloned into pGEM®-T Easy |
| pGEM-cNCED3 | The 1833 bp full-length *VvNCED3* was PCR-amplified from cDNA (using the primers VvNCED5’-ATG and VvNCED3’-STOP) and cloned into pGEM®-T Easy |
